# Author Correction: Atomic-level polarization reversal in sliding ferroelectric semiconductors

**DOI:** 10.1038/s41467-024-48945-3

**Published:** 2024-05-23

**Authors:** Fengrui Sui, Haoyang Li, Ruijuan Qi, Min Jin, Zhiwei Lv, Menghao Wu, Xuechao Liu, Yufan Zheng, Beituo Liu, Rui Ge, Yu-Ning Wu, Rong Huang, Fangyu Yue, Junhao Chu, Chungang Duan

**Affiliations:** 1 Key Laboratory of Polar Materials and Devices (MOE), School of Physics and Electronic Science, East China Normal University, Shanghai, 200062 China; 2 National Key Laboratory of Materials for Integrated Circuits, Shanghai Institute of Microsystem and Information Technology, Chinese Academy of Sciences, Shanghai, 200050 China; 3 College of Materials, Shanghai Dianji University, Shanghai, 201306 China; 4 School of Physics, Huazhong University of Science and Technology, Wuhan, 430074 China; 5 Shanghai Institute of Ceramics, Chinese Academy of Sciences, Shanghai, 200050 China; 6 Collaborative Innovation Center of Extreme Optics, Shanxi University, Taiyuan, Shanxi 030006 China; 7 Shanghai Center of Brain-inspired Intelligent Materials and Devices, East China Normal University, Shanghai, 200062 China; 8 National Laboratory of Infrared Physics, Shanghai Institute of Technical Physics, Shanghai, 200083 China

**Keywords:** Ferroelectrics and multiferroics, Two-dimensional materials

Correction to: *Nature Communications* 10.1038/s41467-024-48218-z, published online 07 May 2024

The original version of this Article contained an error in Fig. 3f, in which the layer stacking mode incorrectly read ‘ABC’ rather than the correct ‘ACB’. This has been corrected in both the PDF and HTML versions of the Article.

