# Supplementary material for: Atomic-level polarization reversal in sliding ferroelectric semiconductors
Source: Nat Commun. 2024 May 7;15:3799. doi: 10.1038/s41467-024-48218-z (PMC11076638; doi:10.1038/s41467-024-48218-z)
Supplement: Supplementary file 1 — Supplementary Information [file 41467_2024_48218_MOESM1_ESM.pdf]

Supplementary Materials

Atomic-level polarization reversal in sliding ferroelectric semiconductors

Fengrui Sui[1], Haoyang Li[1], Ruijuan Qi[1,2*], Min Jin[3*], Zhiwei Lv[1], Menghao Wu[4], Xuechao Liu[5], Yufan Zheng[1], Beituo Liu[1], Rui Ge[1], Yu-Ning Wu[1*], Rong Huang[1], Fangyu Yue[1,6,7*], Junhao Chu[1,8] & Chungang Duan[1,6,7]

[1]Key Laboratory of Polar Materials and Devices (MOE), School of Physics and Electronic Science, East China Normal University, Shanghai 200062, China.

[2]National Key Laboratory of Materials for Integrated Circuits, Shanghai Institute of Microsystem and Information Technology, Chinese Academy of Sciences, Shanghai 200050, China.

[3]College of Materials, Shanghai Dianji University, Shanghai 201306, China.

[4]School of Physics, Huazhong University of Science and Technology, Wuhan 430074, China

[5]Shanghai Institute of ceramics, Chinese Academy of Sciences, Shanghai 200050, China.

[6]Collaborative Innovation Center of Extreme Optics, Shanxi University, Taiyuan, Shanxi 030006, China.

[7]Shanghai Center of Brain-inspired Intelligent Materials and Devices, East China Normal University, Shanghai 200062, China.

[8]National Laboratory of Infrared Physics, Shanghai Institute of Technical Physics, Shanghai 200083, China.

*Correspondence to: rjqi@ee.ecnu.edu.cn (R.Q.). jmaish@aliyun.com (M.J.). ynwu@phy.ecnu.edu.cn (Y.W.). fyyue@ee.ecnu.edu.cn (F.Y.)

Supplementary Methods

1. SHG characterization

SHG measurements were performed at room temperature using a confocal microscope setup in reflection geometry. A mode locked Ti:sapphire laser (Tsunami) at 800 nm (pulse width 100 fs, repetition frequency 80 MHz) was used as the excitation light source. The collimated laser beam was passed through a Glan Thompson polarizer (GTH10M-AM-A, Thorlabs) followed by a wave plate (AQWP05M-600, Thorlabs), which was focused by a ×50 objective lens (Zeiss, 0.75 NA) onto the sample after passing through a beam splitter (CCM1-BS013/M, Thorlabs). The SHG signal was collected by the same objective and directed through a dichroic beam splitter to a spectrometer equipped with a 300 grooves/mm grating and a nitrogen-cooled silicon charge-coupled device (CCD).

2. Derivation of d_{33}^{eff}-values

To quantitatively obtain the piezoelectric information, the contact resonance of the probe can be reduced to a Simple Harmonic Oscillator (SHO) model using IGOR Pro (Asylum Research). The inverse of the optical lever sensitivity (InvOLS) of the probe is calibrated before the experiment to convert the voltage to displacement. During PFM measurements, the experimental parameters used for γ-InSe:Y were V_{ac}=0.5 V, and the range interval of V_{dc} was continuously increased from –3 ~ 3 V to –7 ~ 7 V. The d_{33}^{eff}-value in the weak-indentation limit satisfies the relation with d_{33}, $d_{33}^{eff}=0.5d_{33}$.

Supplementary Text

Supplementary Note 1: The TEM lamella preparation by using FIB and the TEM observation of the 2D sliding structures

FIB is the commonly-used technique for the cross-sectional TEM sample preparation. The FIB lamella generally has a thickness of ~20 - 50 nm, and the depth is about 2 ~ 3 μm with a width of ~3 - 5 μm, giving an observable area (cross-section corresponding to the side-view for layered structures) of ~10 - 15 μm^2, exactly on which the electron-beam of the TEM system illuminates. As shown in Supplementary Fig.1a, 2D vdW-layered materials possess relatively low stiffness, especially the bi- or few-layers with very limited thickness transferred on a substrate. Thus, to obtain a flat lamella with a specific zone axis that is suitable for the HRTEM or HAADF-STEM observations is difficult. And, more seriously, this makes it almost impossible to apply the bias on the ultrathin layers, intrinsically limiting the in-situ observation

and investigation of the atomic arrangement changes involving the external electrical field-induced sliding and resulted polarization reversal in vdW-layered ferroelectrics, since it requires that the FIB-prepared cross-sectional lamellae have a relatively flat and even area (Supplementary Fig.1b), which is several orders of magnitude beyond the atomic-level bi-/few-layer thickness (including twisted moiré structures).

To avoid the potential strain or stress from the probe contact, the upper protective layer (Pt/W) in the FIB-fabricated lamellae is retained as the electrode for probe contact in the in-situ basing TEM system. During the measurements, the probe is force-optimized and kept stable to contact the electrode without direct contact with the sample. The atomic-level observation for the switching is performed at different locations with a certain distance away from the probe.

Basically, there are two kinds of sliding ferroelectric structures. One is the twisted moiré systems[1-3] or heterostructures with defined 3R symmetry[4] with atomic/nanometer thickness. The other is sliding semiconductor systems including MoS_2[5], InSe[6], and GaSe[7]. The moiré superlattices formed in the twisted stacks of vdW crystals exhibit polar domains alternating in moiré length with anti-aligned dipoles (featuring moiré domain antiferroelectric arrangement), showing dissimilar domain dynamics[8]. To reveal the sliding dynamics in twisted interfaces (including twisted moiré structures), it mainly depends on the observation of the twisted lattice induced antiferroelectric moiré domains alternative changing behavior (indirect phenomena related to the domain wall motion) by the top-view observation by using dark field TEM[8] or scanning electron microscopy (SEM) based on the channelling effect[9] or KPFM[2] at micrometer-scale, under the influence of external electric fields. And, due to the domain antiferroelectric arrangement feature, the voltage used for polarization reversal (domain wall motion) for so thin layered moiré systems is obviously high (±30V[8] or ~2.2 V/nm[9]), which is undesired for low-energy nanodevices. Also, as reported, as the domain wall pinning due to structural disorder, such as rough edges, bubbles or disorders in the adjacent layer formed during moiré superlattice preparation[8], it is not easy to precisely monitor and manipulate the dynamic behavior and the accompanying charge transfer in moiré superlattice system. In contrast, sliding ferroelectric semiconductors provide a suitable platform for exploring the interlayer sliding induced polarization reversal[6].

Supplementary Note 2: Details of the Y-related defect calculation in InSe:Y

Three types of Y-related defects including Y interstitial (Y_i), Y substituting Se (Y_{Se}) and Y substituting In (Y_{In}) are considered. For both Y_{Se} and Y_{In}, two inequivalent structures exist due to the symmetry, which sit in the upper or lower half within one single layer of InSe. Due to

the relatively weak vdW interaction, the calculated formation energies for the equivalent structures are almost degenerate. The most stable Y_i is a Y atom sitting in the vdW-gap as an interlayer bridge (Fig.1c). The calculated formation energies of the three defects under both In-rich and In-poor conditions (Fig.1d) demonstrate that i) the most stable Y_i with +2 charge state has the lowest formation energies for the whole range of the bandgap; ii) the Y_{In} is more stable with +2 charge state if the Fermi level (E_F) is close to the valence band (VB), whereas the neutral state becomes more stable if the E_F is higher than 0.48 eV above the VB maximum (VBM); iii) the formation energies of Y_{In} and Y_i with the most stable charge states are almost equal if the E_F is reaching the conduction band maximum (CBM); and iv) the formation energy of Y_{Se} is much higher than the other two defects.

To evaluate the effect of Y-doping on the sliding barriers, three different Y_i positions are considered (positions 1, 2 and 3 in Supplementary Fig.14). Three configurations exhibit similar barrier heights, which are around 31.0 meV per f.u. (31.08 meV, 31.14 meV and 30.92 meV for positions 1, 2 and 3, respectively) from the initial ABC-stacking structure. Due to the symmetry, the intermediate state, or the ε-phase, has the same energy for three doping positions.

Supplementary Note 3: Effect of Y interstitial on the sliding of bilayer system

To realize the polarization reversal, there are two possible paths with different sliding directions, denoted as the short path and the long path (as shown in Supplementary Fig.3a and b). The short path covers 1/3 of the total sliding distance of an entire period along the sliding distance, while the long path covers 2/3. The barriers of two sliding directions are plotted included in Fig.1e, as well as the barriers with Y interstitial in between the layers. The shaded area means sliding along the short path.

Before Y doping, the sliding prefers the short path because of lower barrier (blue curve), while the sliding along the long path seems to have higher barrier. This is because the long-path sliding needs to overcome a barrier corresponding to the “AA stacking”, and the short-path sliding does not.

With Y interstitial, sliding barriers significantly increase for both sliding directions (red curve), and the short path still has lower barrier than the long path. To mimic the sliding in the bulk system, the interlayer vdW gap is maintained during the sliding. We need to emphasize that the short path is not adopted in the discussion of polarization reversal in bulk InSe and InSe:Y. In bulk system, the short-path sliding in bilayer system cannot achieve the reversal for the whole bulk, because the barrier is caused by both upper and lower layers.

Supplementary Note 4: Sliding patterns in bulk system

The proposed sliding patterns in Fig.4d are all based on the long path sliding as mentioned in Supplementary Note 3 and Supplementary Fig.3a and c. For example, the sliding pattern for Path 1 in Fig.4d is the relative sliding of the middle and the bottom layers along the long path as mentioned in Supplementary Note 3. We also considered a possible sliding path that the middle layer B slides towards left to C and the bottom layer C also slides towards left to A and then B. NEB calculation shows a barrier of 44.5 eV per f.u., which is higher than the 37.2 eV per f.u. of the relative sliding as we adopted.

Supplementary Note 5: Optimizing the cycling and retention feature of ferroelectric InSe:Y during PFM characterization

PFM is a powerful technique for the characterization of ferroelectric materials by measuring the dynamic electromechanical response[10]. For 2D vdW-layered ferroelectrics, it is worth noting that the PFM measurements are easier to be influenced, especially when the thickness is continuously decreased, possibly due to the voltage-induced thermal accumulation effect and the charging or contamination at the interface between the narrow-gap InSe:Y flake (with an optical bandgap of ~1.2 eV and a high mobility of 10^3 $cm^2/V\cdot s$)[11,12] and the substrate. The weakened signal after more cycles also supports this assumption (Supplementary Figs.5 and 4d,e).

To verify the intrinsic ferroelectricity of InSe:Y, we optimize the sample preparation by introducing fresh exfoliated multilayer graphene under the InSe:Y flake (Supplementary Fig.4a,b) to reduce the interface issues (such as charging or contaminations from the substrate), which shows excellent cycling stability (Supplementary Fig.4b,c,f,g) during the PFM characterization and ferroelectric stability even after ~83 days (Supplementary Fig.4h). These excellent cycling stability and good retention feature show great application potential in the future nanoelectronics.

Supplementary Note 6: Simulation on the structure models and SAED/FFT patterns of InSe:Y before and after OOP polarization switching

Different from the traditional ferroelectrics, the sliding ferroelectricity exclusively exists in vdW-layered materials with the polarization switching induced by interlayer sliding, which will not exhibit obvious domain formation or motion that can be observed by using traditional dark field TEM mode. So we perform detailed simulation of the polarization switching states for ferroelectric InSe:Y (Supplementary Fig.9), which shows that the OOP polarization

switching will result in a mirrored SAED pattern (Supplementary Fig.9d) compared with the pristine one (Supplementary Fig.9c). If two OOP polarization states present in the same region, two sets of mirrored SAED or FFT patterns will be obtained. It is just like what we have observed in the FFT patterns for the snapshots during in-situ biasing (Supplementary Fig.8). Furthermore, subsequent reduced FFT analyses for the HAADF images taken after in-situ biasing confirm the co-existence of two OOP polarization states (Fig.3c and Supplementary Fig.11) in the nanometer level, as all the single layer in the HAADF images possessing same atomic configuration will not show in-plane inversion induced FFT reversal.

Supplementary Note 7: Ripplocation and polarization analyses in InSe:Y after in-situ biasing TEM observation

To obtain the change of interlayer spacing due to ripplocations, we perform detailed atomic arrangement analyses for HAADF-STEM images combining with atomic quantification via Calatom software based on custom MATLAB scripts[13]. As shown in Fig.3b, the change of interlayers is straight, narrow, and crystallographically oriented, in accord with the characteristics of ripplocations[14]. And, we explicitly measure this distance change of the interlayer spacing due to the ripplocations, showing an average value of about 36 pm (varying within 20 pm ~ 50 pm), which is comparable to the reported value in transition metal dichalcogenides both theoretically and experimentally[14], which further confirms the ripplocation features, different from mechanical delamination or peelings of the lamella due to the field-induced or mechanical strain.

For the polarization analyses, we mark the polarization direction (pointing from anion to cation), without the polarization strength information.

Supplementary Figures

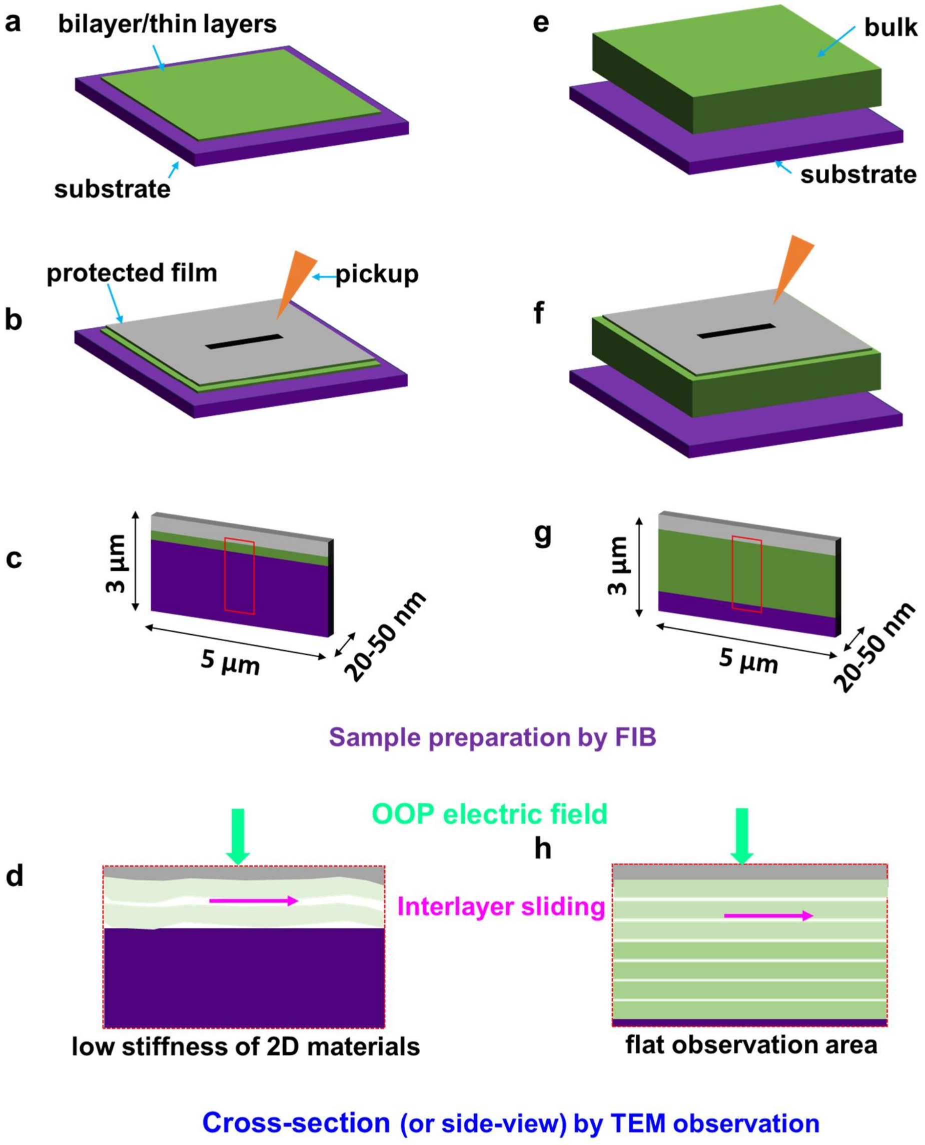

Supplementary Fig.1 Schematics of FIB-fabricated samples for TEM observation. **a – d**, For ultrathin 2D vdW-layered materials. **e – h,** For thick or bulk 2D vdW-layered materials.

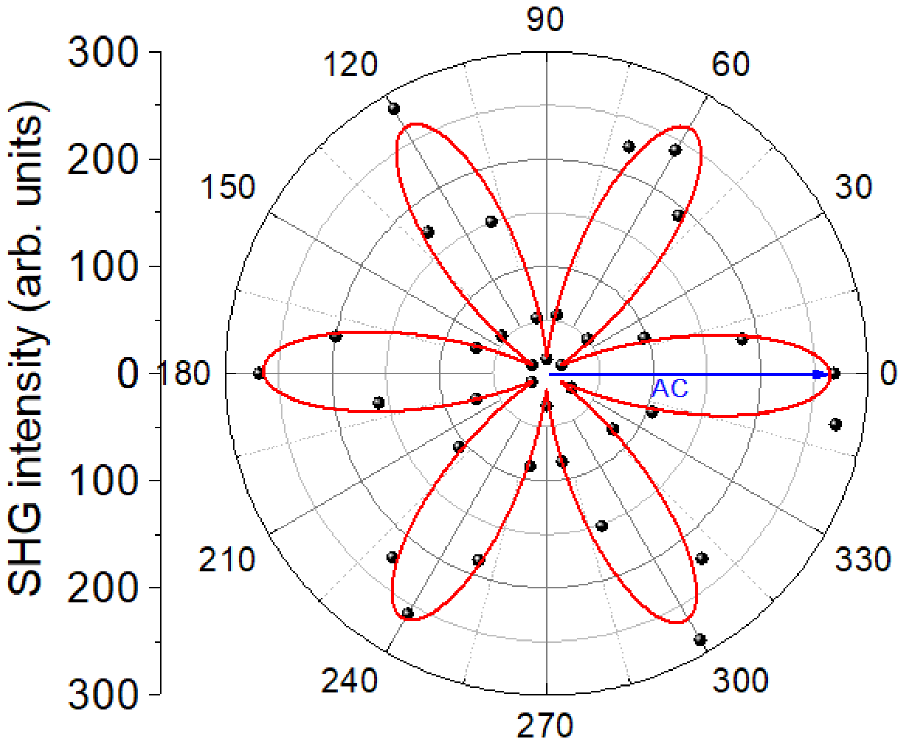

Supplementary Fig.2 SHG results of InSe:Y. Polar plots of SHG intensities collected by rotating the polarizer as a function of the detection angle.

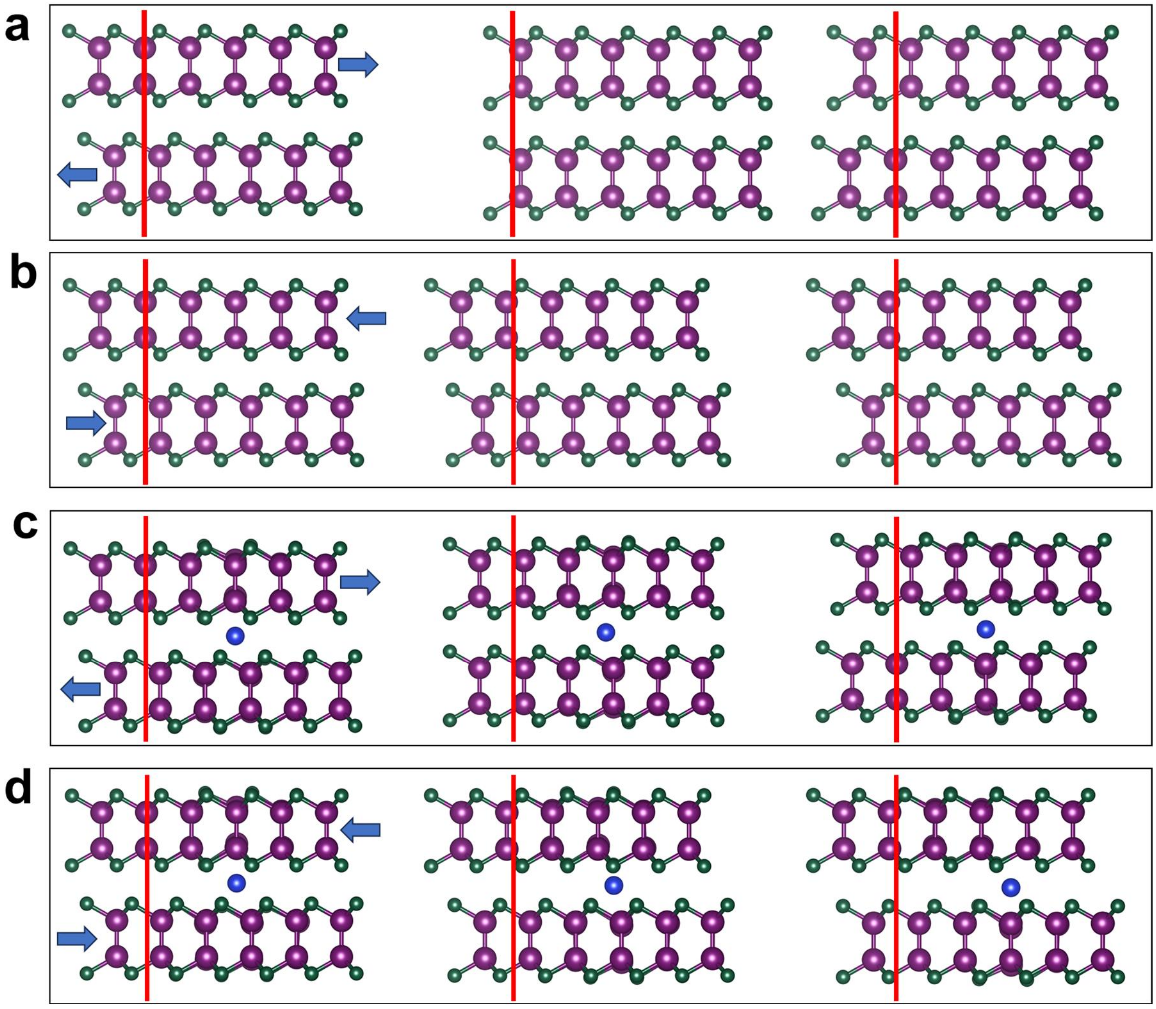

Supplementary Fig.3 Schematics of interlayer sliding for pristine and Y-doped InSe bilayer. a, Long-path sliding in InSe bilayer. **b**, Short-path sliding in InSe bilayer. **c**, Long-path sliding in InSe:Y bilayer. **d**, Short-path sliding in InSe:Y bilayer.

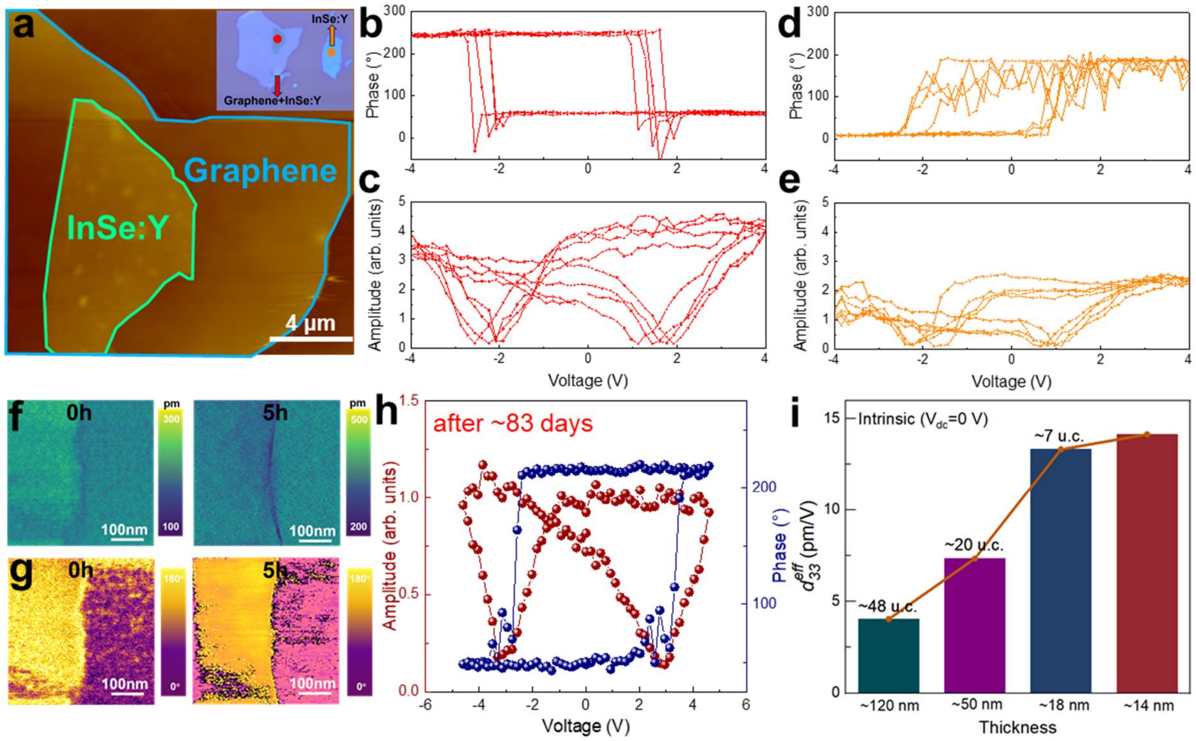

Supplementary Fig.4 PFM characterization in InSe:Y. **a**, The AFM image of InSe:Y/graphene. The inset is the optical microscopic image of InSe:Y flakes with/without the bottom graphene for PFM measurement. **b – e,** The local PFM phase (**b** and **d**) and amplitude (**c** and **e**) loops during the polarization switching process of the InSe:Y flake in **a**. **f, g**, The amplitude (**f**) and phase (**g**) images after domain writing. **h,** The local PFM amplitude and phase loops of the flake in **a** after ~83 days, showing the stable ferroelectricity in InSe:Y. **i**, The d_{33}^{eff}-value for InSe:Y flakes with different thicknesses from ~14 nm (~5 u.c.) to 120 nm (~48 u.c.).

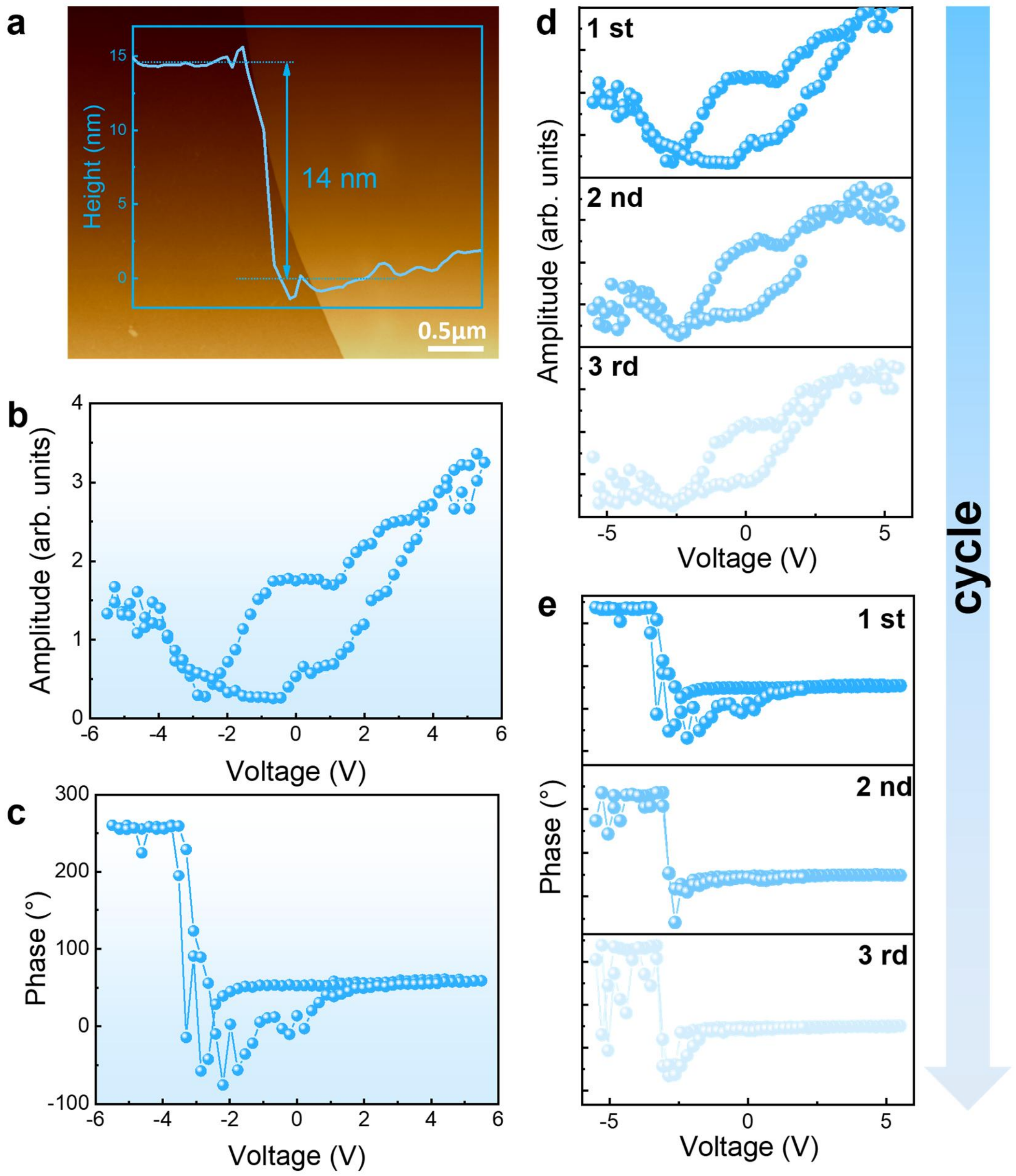

Supplementary Fig.5 Cycling feature of the PFM results in a 14 nm (~5 u.c.) InSe:Y flake. **a**, AFM image. **b, c**, The local PFM amplitude (**b**) and phase (**c**) loops during the polarization switching process of the InSe:Y flakes in **a**. **d, e,** The repeated PFM results (three cycles) of the ~14 nm flake.

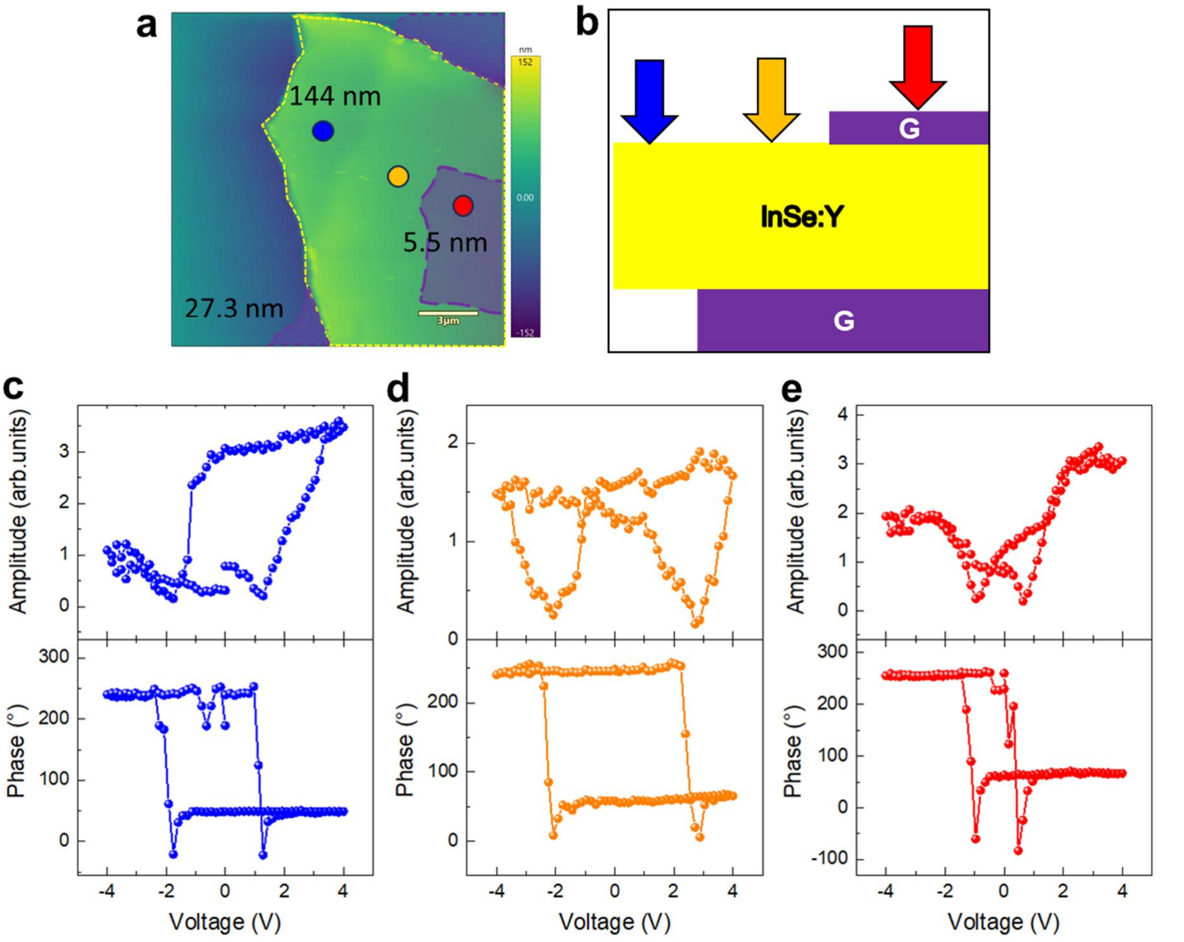

Supplementary Fig.6 PFM results for different kinds of InSe:Y stacked devices. **a**, AFM image. **b**, Schematic of different InSe:Y/graphene stacked devices. **c – e,** Local PFM amplitude and phase loops during the polarization switching process of the InSe:Y/Si device (**c**), InSe:Y/graphene/Si device (**d**), and graphene/InSe:Y/graphene/Si device (**e**).

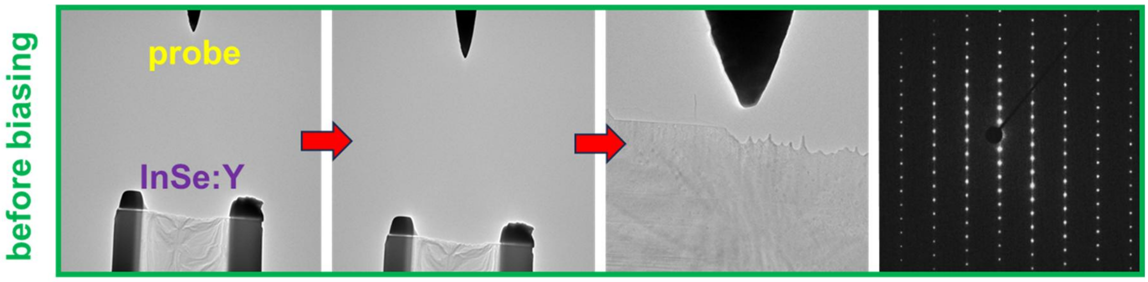

Supplementary Fig.7 In-situ biasing procedure performed on a Cs-TEM system. Low-magnified TEM images and the corresponding SAED pattern taken before biasing.

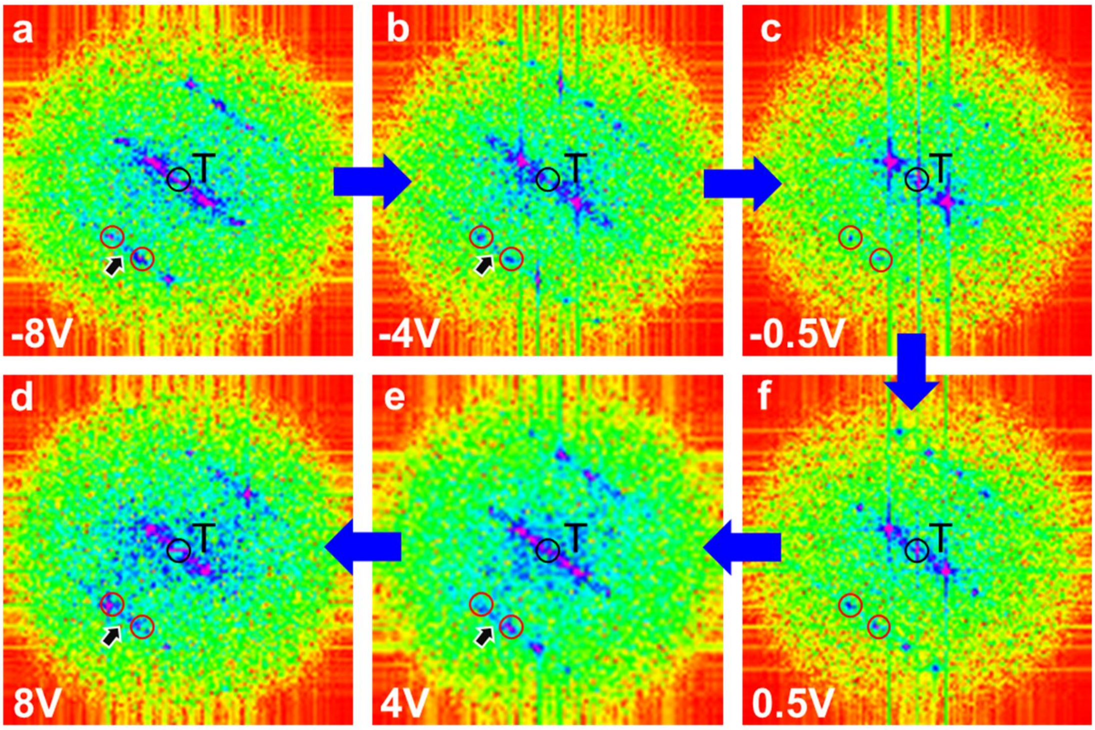

Supplementary Fig.8 The FFT patterns of the HRTEM snapshots. a – f, corresponding to those in Fig.2b.

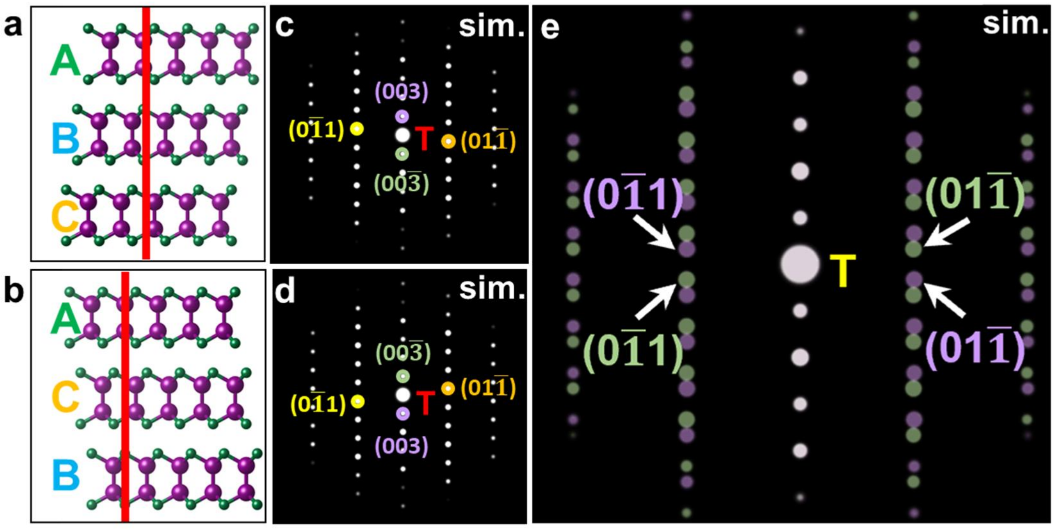

Supplementary Fig.9 Structure simulation of the polarization switching. Structure models (**a** - **b**) of interlayer sliding induced reversed OOP polarization states and simulated SAED patterns (**c** - **d**). **e**, Simulated SAED patterns taken at the area with the co-existence of two reversed OOP polarization states.

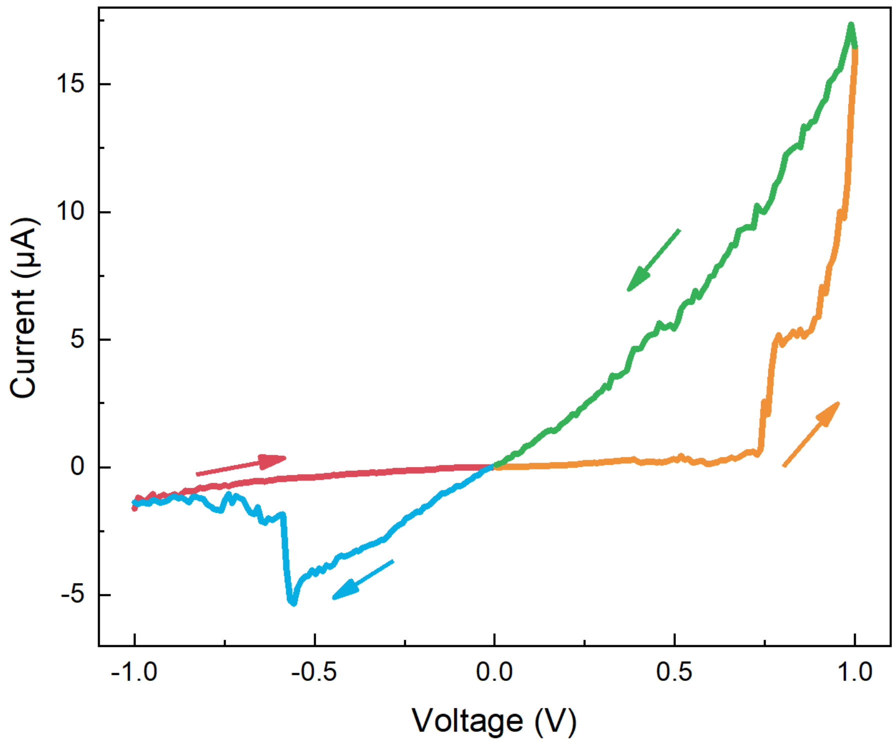

Supplementary Fig.10 I-V curve during the in-situ biasing TEM measurement.

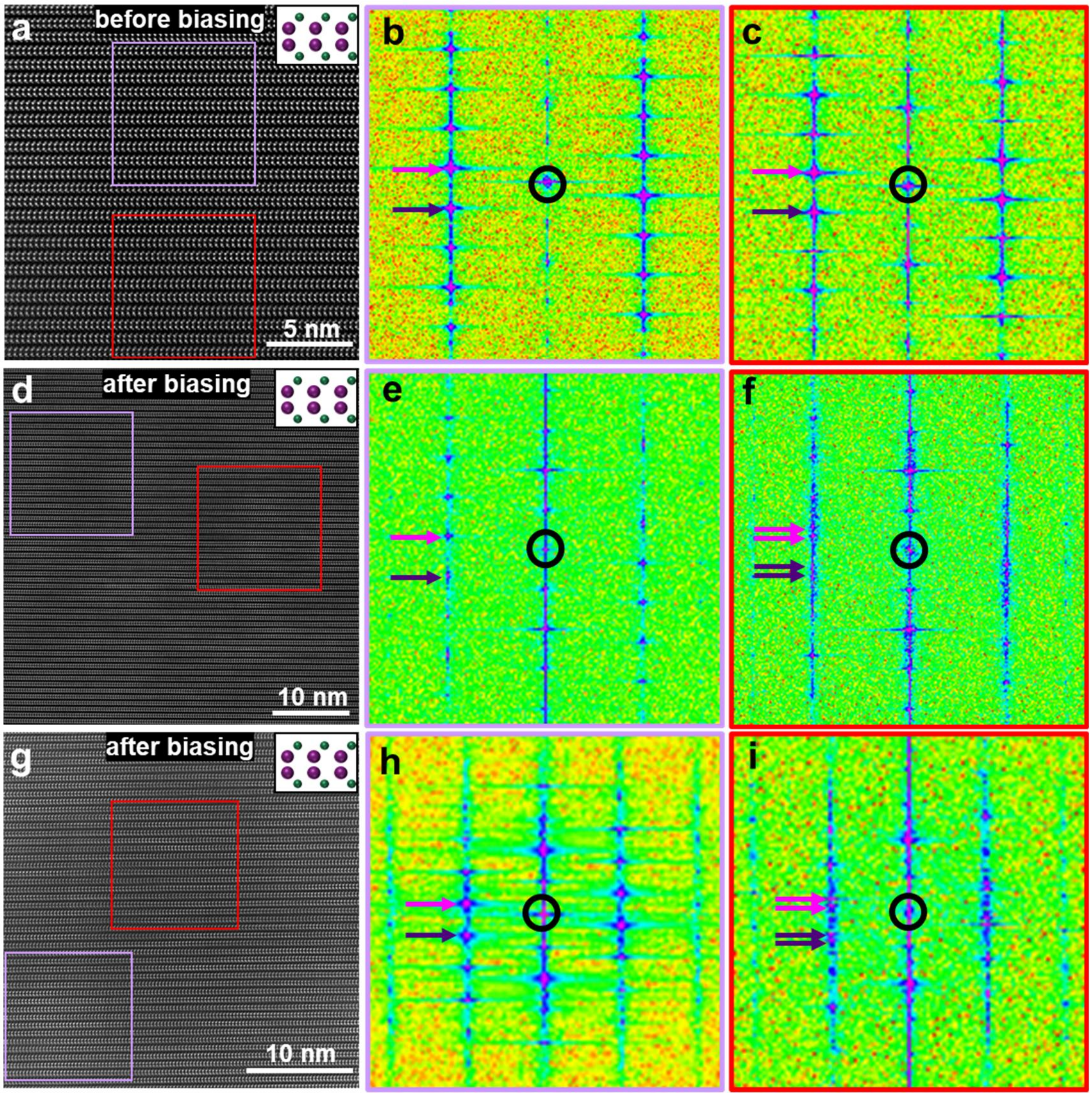

Supplementary Fig.11 Additional atomic image analyses of polarization switching by in-situ biasing. **a**, HAADF-STEM images taken at different areas with the corresponding reduced FFT patterns (**b** - **c**) of InSe:Y specimen without biasing (i.e., before in-situ biasing), showing the uniform atomic arrangement (or polarization state). **d** – **g,** HAADF-STEM images taken at different areas with the corresponding reduced FFT patterns (**e** - **f**, and **h** - **i**) of InSe:Y specimen after in-situ biasing, showing different polarization states. Note: **b** and **c** separately for the purple and red areas marked in **a**, **e** and **f** separately for those in **d**, and **h** and **i** separately for those in **g**.

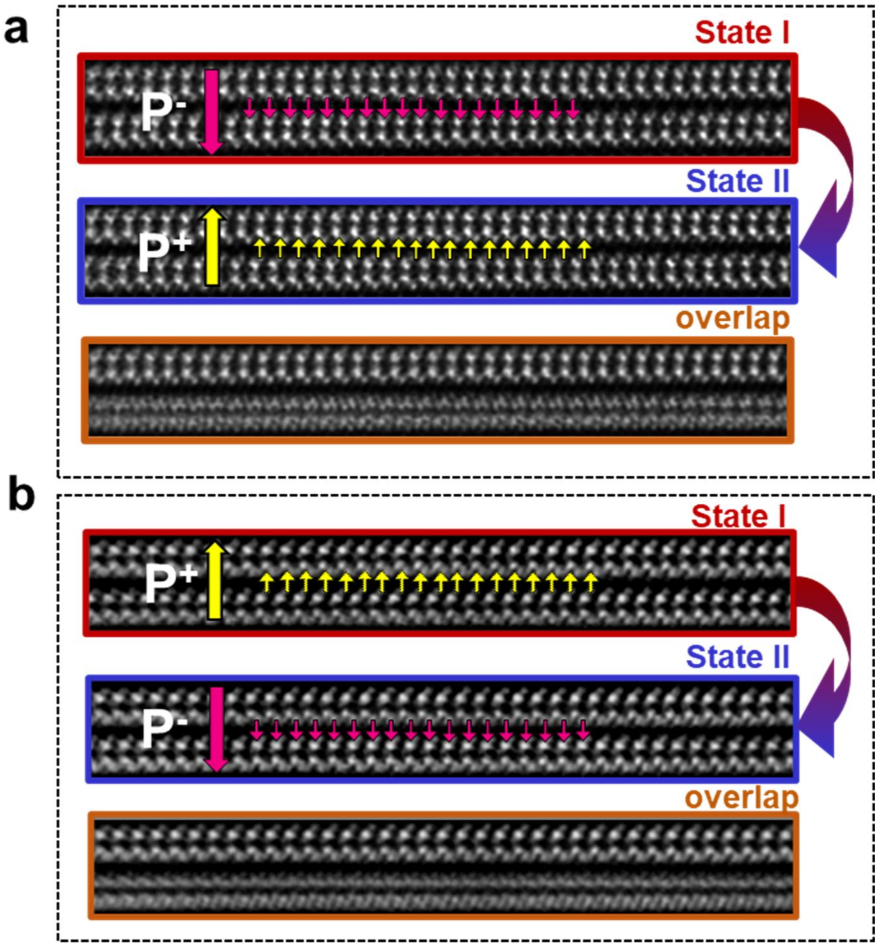

Supplementary Fig.12 Atomic-level polarization switching. **a,b,** Atomic-scale single layer sliding-induced OOP polarization switching at initial (state I), final (state II) and overlapped states. The atomic interlayer sliding can be observed exclusively in different ferroelectric InSe:Y samples but cannot be observed in undoped InSe samples.

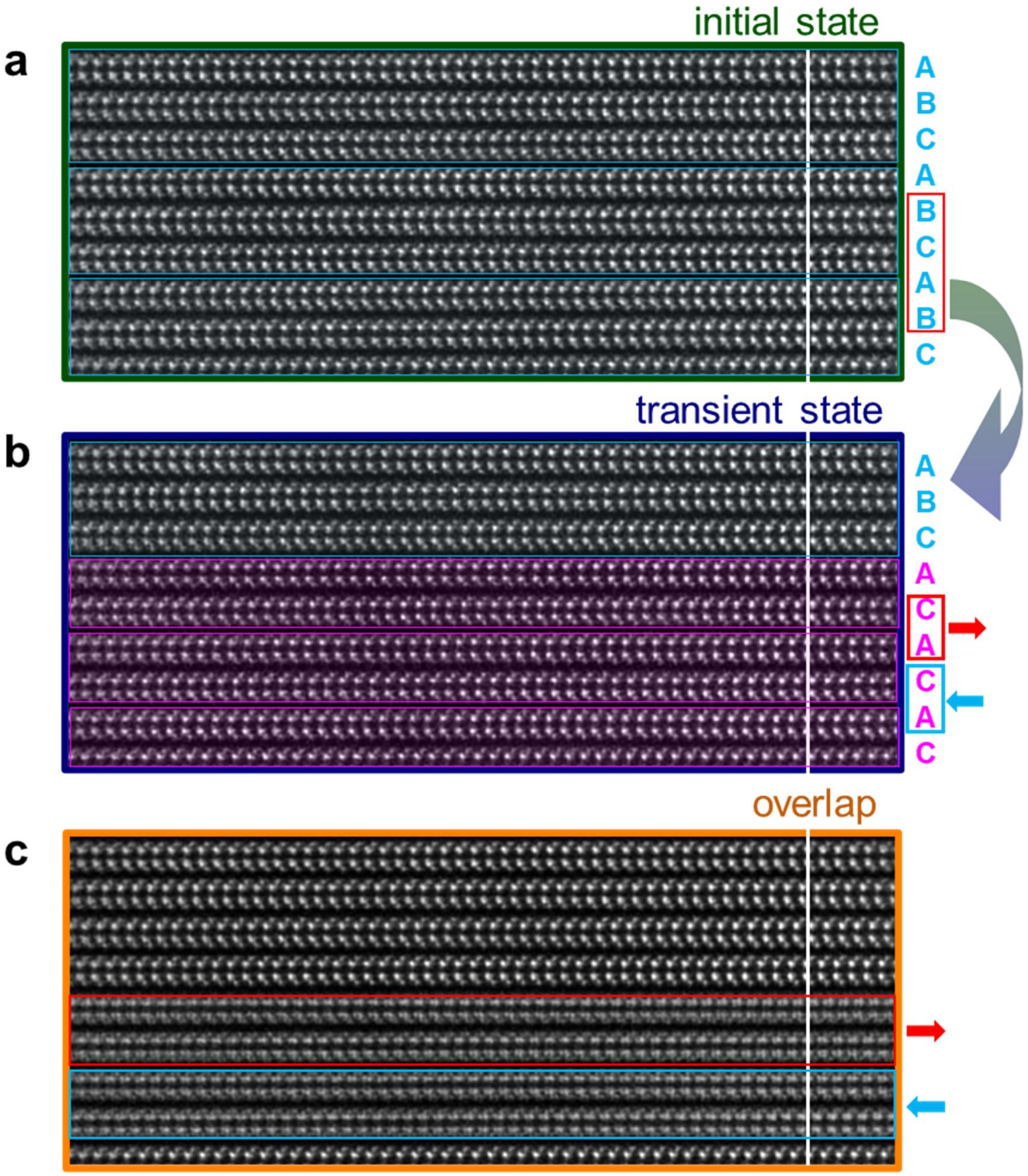

Supplementary Fig.13 Additional atomic-level polarization switching. **a**, The initial state of the atomic-scale arrangement with the ABCABC stacking style (i.e., γ-InSe). **b**, The sliding-induced intermediate state with ACACAC-stacking (i.e., ε-InSe). **c**, The overlapped state to show the electric field-induced sliding dynamics.

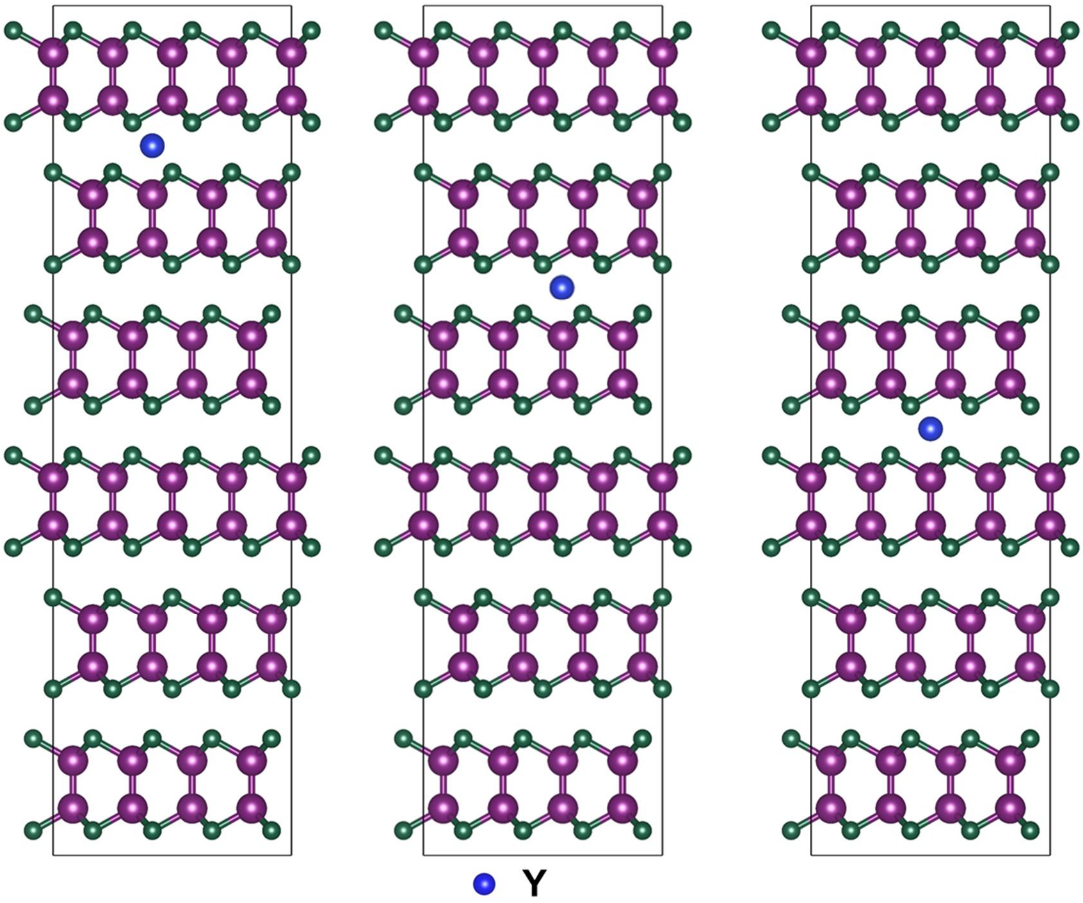

Supplementary Fig.14 The doped-Y siting at different interlayer interstitial positions in InSe for theoretical calculation.

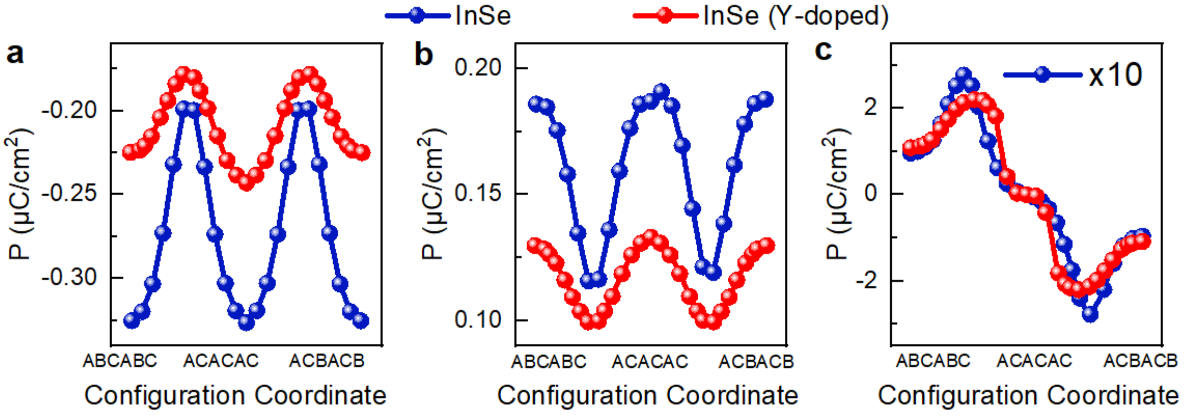

Supplementary Fig.15 The polarizations as well as their evolution along the sliding paths for γ-InSe before and after Y-doping. **a**, x direction (zigzag). **b**, y direction (armchair). **c**, z direction (OOP).

Supplementary Tables

Supplementary table 1. Calculated polarization along different directions in pristine or Y-doped InSe. The unit is μC/cm^2.

vdW-layered InSe		x (zigzag)	y (armchair)	z (OOP)
undoped	ABC	-0.325	0.186	0.096
	ACB	-0.325	0.186	-0.096
Y-doped	ABC	-0.225	0.130	0.931
	ACB	-0.225	0.130	-0.931

Supplementary table 2. A comparative context of OOP polarization and energy barrier of 2D vdW ferroelectric systems

Materials	Form	Vertical polarization	Energy barrier
(15-crown-5) Cd_3Cl_6	bulk[15]	~0.4 $\mu C/cm^2$	0.62 meV lower for 88 atoms
Bi_2Te_3	trilayer[16]	β_1-Bi_2Te_3: -5.1×10^9 e/cm^2	69.46 meV/u.c.
		β_2-Bi_2Te_3: 5.1×10^9 e/cm^2	
h-BN	bilayer[2]	1.1 nm^2: 0.55 Debye/nm^2	\
		2.9 nm^2: 0.45 Debye/nm^2	
	bilayer[17]	\	ThBN/G: 2 meV/nm^2
			hBN/G: 80 meV/nm^2
	bilayer[18]	P_{2D}: 2.08 pC/m	~9 meV/u.c.
		P_{3D}: 0.68 $\mu C/cm^2$	
	bilayer[1]	P_{2D}: 2.25 pC/m	Exp.: 0.15 meV/u.c.
		P_{3D}: 0.68 $\mu C/cm^2$	The.: 9 meV/u.c.
BP<br>BAs<br>BSb	bilayer[19]	1.075 pC/m	3 meV/u.c.
		0.965 pC/m	12 meV/u.c.
		3.707 pC/m	195 meV/u.c.
	bilayer[20]	1.6 pC/m	19.5 meV/u.c.
		2.2 pC/m	28.4 meV/u.c.
		13.9 pC/m	128.5 meV/u.c.
CrI_3<br>$Cr_2Ge_2Te_6$<br>Fe_3GeTe_2	bilayer[21]	0.18 pC/m	23 meV/f.u.
		0.209 pC/m	58 meV/f.u.
		0.1121 pC/m	20 meV/f.u.
T-$FeCl_2$	bilayer[22]	state I: −2.7 nC/cm^2	4.2 meV/f.u.
		state II: 2.7 nC/cm^2	
MX<br>(M = Ge, Sn;<br>X = S, Se)	bilayer[23]	≥ 40 $\mu C/cm^2$	GeS: 78 meV/atom
			GeSe: 33 meV/atom
			SnS: 0.1 meV/atom
			SnSe: 1.3 meV/atom
	γ-GeSe[24] bulk<br>bilayer	0.296 $\mu C/cm^2$	71 meV/u.c.
		0.471 pC/m	19 meV/u.c.
3R-$LaBr_2$	bilayer[25]	116 $\mu C/m^2$	14.81 meV/u.c.
2H-$MnBi_2Te_4$	bilayer[26]	AB’: 0.636 pC/m	30 meV/u.c.
		AC’: −0.647 pC/m	
MnSe	bilayer[27]	2.7 pC/m	8.4 meV/f.u.
	trilayer[27]	6.7 pC/m	12 meV/f.u.
	tetralayer[27]	10.6 pC/m	13 meV/f.u.
ZrI_2	trilayer[28]	$T_0 \rightarrow 1T'$: \	$T_0 \rightarrow 1T'$: 5.3 meV/u.c.
		$T_0 \rightarrow T_d$: 0.243 $\mu C/cm^2$	$T_0 \rightarrow T_d$: <50 μeV/u.c.
	bilayer[29]	210 $\mu C/m^2$	180°: 1.6 meV/f.u.
			120°: 0.07 meV/f.u.

Materials	Form	Vertical polarization	Energy barrier
phosphorene<br>arsenene<br>antimonene	bilayer[30]	0.53 pC/m (bulk: 0.09 μC/cm^2)	10 meV/f.u.
		1.33 pC/m (bulk: 0.22 μC/cm^2)	18 meV/f.u.
		1.45 pC/m (bulk: 0.24 μC/cm^2)	38 meV/f.u.
ReS_2	bilayer[31] (0 K)	0.07 pC/m	~17.1 meV/u.c.
MoS_2/WS_2	bilayer[4] (3R-like)	up: 0.60 pC/m	1.9 meV/f.u.
		down: −1.45 pC/m	16 meV/f.u.
3R MoS_2	trilayer[5]	\	path 1: 15.4 meV/f.u.
			path 2: 30.6 meV/f.u.
			path 3: 83.3 meV/f.u.
VS_2	bilayer[32]	2.018×10^{-3} C/m^2	19 meV/u.c.
H-VSe_2	bilayer[33]	±0.078 μC/cm^2	64 meV/f.u.
T'-VTe_2	bilayer[34]	2.84×10^{-4} C/m^2	0.026 eV/f.u.
WTe_2	trilayer[35]	~0.19 μC/cm^2	$P_{up}\rightarrow P_{dn1}$: 0.70 meV/f.u.
			$P_{up}\rightarrow P_{dn2}$: 0.29 meV/f.u.
Sn_2S_3	bilayer[36]	State I: 11.14 pC/m	35.4 meV/f.u.
		State II: 10.81 pC/m	31.1 meV/f.u.
		State III: 4.05 pC/m	12.2 meV/f.u.
$MoSi_2N_4$<br>$MoGe_2N_4$	bilayer[37]	3.36 pC/m	20.20 meV/u.c.
		3.05 pC/m	33.23 meV/u.c.
VSi_2P_4	bilayer[38]	±0.42 pC/m	path I: 9 meV/f.u.
			path II: 37 meV/f.u.
In_2Se_3	bilayer[39]	\	5 meV/f.u.
α-In_2Se_3	monolayer[40]	0.97 μC/cm^2	31.1 meV/f.u.

Supplementary References

1 Yasuda, K., Wang, X., Watanabe, K., Taniguchi, T. & Jarillo-Herrero, P. Stacking-engineered ferroelectricity in bilayer boron nitride. *Science* **372**, 5 (2021). https://doi.org:10.1126/science.abd3230

2 Vizner Stern, M. *et al.* Interfacial ferroelectricity by van der Waals sliding. *Science* **372**, 5 (2021). https://doi.org:10.1126/science.abe8177

3 Zheng, Z. *et al.* Unconventional ferroelectricity in moire heterostructures. *Nature* **588**, 71-76 (2020). https://doi.org:10.1038/s41586-020-2970-9

4 Rogee, L. *et al.* Ferroelectricity in untwisted heterobilayers of transition metal dichalcogenides. *Science* **376**, 973-978 (2022). https://doi.org:10.1126/science.abm5734

5 Meng, P. *et al.* Sliding induced multiple polarization states in two-dimensional ferroelectrics. *Nat. Commun.* **13**, 7696 (2022). https://doi.org:10.1038/s41467-022-35339-6

6 Sui, F. *et al.* Sliding ferroelectricity in van der Waals layered gamma-InSe semiconductor. *Nat. Commun.* **14**, 36 (2023). https://doi.org:10.1038/s41467-022-35490-0

7 Li, W. H. *et al.* Emergence of ferroelectricity in a nonferroelectric monolayer. *Nat. Commun.* **14**, 2757 (2023). https://doi.org:10.1038/s41467-023-38445-1

8 Ko, K. *et al.* Operando electron microscopy investigation of polar domain dynamics in twisted van der Waals homobilayers. *Nat. Mater.* **22**, 992-998 (2023). https://doi.org:10.1038/s41563-023-01595-0

9 Weston, A. *et al.* Interfacial ferroelectricity in marginally twisted 2D semiconductors. *Nat. Nanotechnol.* **17**, 390-395 (2022). https://doi.org:10.1038/s41565-022-01072-w

10 Guan, Z. *et al.* Identifying intrinsic ferroelectricity of thin film with piezoresponse force microscopy. *AIP Adv.* **7**, 095116 (2017). https://doi.org:10.1063/1.4999199

11 Yuan, K. *et al.* Realization of quantum Hall effect in chemically synthesized InSe. *Adv. Funct. Mater.* **29**, 1904032 (2019). https://doi.org:10.1002/adfm.201904032

12 Bandurin, D. A. *et al.* High electron mobility, quantum Hall effect and anomalous optical response in atomically thin InSe. *Nat. Nanotechnol.* **12**, 223-227 (2017). https://doi.org:10.1038/nnano.2016.242

13 Zhang, Q., Zhang, L. Y., Jin, C. H., Wang, Y. M. & Lin, F. CalAtom: A software for quantitatively analysing atomic columns in a transmission electron microscope image.

Ultramicroscopy **202**, 114-120 (2019). https://doi.org:10.1016/j.ultramic.2019.04.007

14 Kushima, A., Qian, X., Zhao, P., Zhang, S. & Li, J. Ripplocations in van der Waals layers. *Nano Lett.* **15**, 1302-1308 (2015). https://doi.org:10.1021/nl5045082

15 Miao, L.-P. et al. Direct observation of geometric and sliding ferroelectricity in an amphidynamic crystal. *Nat. Mater.* **21**, 1158-1164 (2022). https://doi:10.1038/s41563-022-01322-1

16 Liang, Y. et al. Intertwined ferroelectricity and topological state in two-dimensional multilayer. *npj Comput. Mater.* **7**, 172 (2021). https://doi:10.1038/s41524-021-00643-0

17 Lv, M. et al. Spatially resolved polarization manipulation of ferroelectricity in twisted hBN. *Adv. Mater.* **34**, 2203990 (2022). https://doi:10.1002/adma.202203990

18 Li, L. & Wu, M. Binary compound bilayer and multilayer with vertical polarizations: Two-dimensional ferroelectrics, multiferroics, and nanogenerators. *ACS Nano* **11**, 6382-6388 (2017). https://doi:10.1021/acsnano.7b02756

19 Wang, Z., Gui, Z. & Huang, L. Sliding ferroelectricity in bilayer honeycomb structures: a first-principles study. *Phys. Rev. B* **107**, 035426 (2023). https://doi:10.1103/PhysRevB.107.035426

20 Wang, Q. et al. Universal co-existence of photovoltaics and ferroelectricity from a two-dimensional 3R bilayer BX (X = P, As, Sb). *J. Mater. Chem. C* **10**, 1048-1061 (2022). https://doi:10.1039/d1tc03166j

21 Zhong, T., Cheng, L., Ren, Y. & Wu, M. Theoretical studies of sliding ferroelectricity, magnetoelectric couplings, and piezo-multiferroicity in two-dimensional magnetic materials. *Chem. Phys. Lett.* **818**, 140430 (2023). https://doi:10.1016/j.cplett.2023.140430

22 Zhang, T., Xu, X., Huang, B., Dai, Y. & Ma, Y. 2D spontaneous valley polarization from inversion symmetric single-layer lattices. *npj Comput. Mater.* **8**, 64 (2022). https://doi:10.1038/s41524-022-00748-0

23 Xu, B., Deng, J., Ding, X., Sun, J. & Liu, J. Z. Van der Waals force-induced intralayer ferroelectric-to-antiferroelectric transition via interlayer sliding in bilayer group-IV monochalcogenides. *npj Comput. Mater.* **8**, 47 (2022). https://doi:10.1038/s41524-022-00724-8

24 Kim, H.-G. & Choi, H. J. Quasiparticle band structures, spontaneous polarization, and spin-splitting in noncentrosymmetric few-layer and bulk γ-GeSe. *J. Mater. Chem. C* **9**, 9683-9691 (2021). https://doi:10.1039/d1tc01800k

25 Sun, W. et al. $LaBr_2$ bilayer multiferroic moiré superlattice with robust magnetoelectric

coupling and magnetic bimerons. *npj Comput. Mater.* **8**, 159 (2022). https://doi:10.1038/s41524-022-00833-4

26 Ren, Y., Ke, S., Lou, W.-K. & Chang, K. Quantum phase transitions driven by sliding in bilayer $MnBi_2Te_4$. *Phys. Rev. B* **106**, 235302 (2022). https://doi:10.1103/PhysRevB.106.235302

27 Liu, K., Ma, X., Xu, S., Li, Y. & Zhao, M. Tunable sliding ferroelectricity and magnetoelectric coupling in two-dimensional multiferroic MnSe materials. *npj Comput. Mater.* **9**, 16 (2023). https://doi:10.1038/s41524-023-00972-2

28 Ma, X., Liu, C., Ren, W. & Nikolaev, S. A. Tunable vertical ferroelectricity and domain walls by interlayer sliding in β-ZrI_2. *npj Comput. Mater.* **7**, 177 (2021). https://doi:10.1038/s41524-021-00648-9

29 Zhang, T. et al. Ferroelastic-ferroelectric multiferroics in a bilayer lattice. *Phys. Rev. B* **103**, 165420 (2021). https://doi:10.1103/PhysRevB.103.165420

30 Liang, Y. et al. Out-of-plane ferroelectricity and multiferroicity in elemental bilayer phosphorene, arsenene, and antimonene. *Appl. Phys. Lett.* **118**, 012905 (2021). https://doi:10.1063/5.0032790

31 Wan, Y. et al. Room-temperature ferroelectricity in 1T′-ReS_2 multilayers. *Phys. Rev. Lett.* **128**, 067601 (2022). https://doi:10.1103/PhysRevLett.128.067601

32 Liu, X., Pyatakov, A. P. & Ren, W. Magnetoelectric coupling in multiferroic bilayer VS_2. *Phys. Rev. Lett.* **125**, 247601 (2020). https://doi:10.1103/PhysRevLett.125.247601

33 Zhang, C., Guo, P. & Zhou, J. Tailoring bulk photovoltaic effects in magnetic sliding ferroelectric materials. *Nano Lett.* **22**, 9297-9305 (2022). https://doi:10.1021/acs.nanolett.2c02802

34 Zhang, T., Xu, X., Dai, Y., Huang, B. & Ma, Y. Intrinsic ferromagnetic triferroicity in bilayer T′-VTe_2. *Appl. Phys. Lett.* 120, 192903 (2022). https://doi:10.1063/5.0092079

35 Sharma, P. et al. A room-temperature ferroelectric semimetal. *Sci. Adv.* **5**, eaax5080 (2019). https://doi:10.1126/sciadv.aax5080

36 Li, L. & Wu, M. Vertical ferroelectricity in two-dimensional mixed-valence tin sulfide system: Unprecedented piezoelectricity, efficient nanogenerator and facile control of morphotoropic phase transformations. *Nano Energy* **83**, 105786 (2021). https://doi:10.1016/j.nanoen.2021.105786

37 Zhong, T., Ren, Y., Zhang, Z., Gao, J. & Wu, M. Sliding ferroelectricity in two-dimensional MoA_2N_4(A = Si or Ge) bilayers: high polarizations and Moiré potentials. *J. Mater. Chem. A* **9**, 19659-19663 (2021). https://doi:10.1039/d1ta02645c

38 Zhang, T. et al. Layer-polarized anomalous Hall effects in valleytronic van der Waals bilayers. *Mater. Horiz.* 10, 483-490 (2023). https://doi:10.1039/d2mh00906d

39 Ding, W. et al. Prediction of intrinsic two-dimensional ferroelectrics in In_2Se_3 and other III_2-VI_3 van der Waals materials. *Nat. Commun.* **8**, 14956 (2017). https://doi:10.1038/ncomms14956

40 Jiang, X., Feng, Y., Chen, K.-Q. & Tang, L.-M. The coexistence of ferroelectricity and topological phase transition in monolayer α-In_2Se_3 under strain engineering. *J. Phys.: Condens. Matter* **32**, 105501 (2020). https://doi:10.1088/1361-648X/ab58f1
