# Supplementary material for: Atomic-level polarization reversal in sliding ferroelectric semiconductors
Source: Nat Commun. 2024 May 7;15:3799. doi: 10.1038/s41467-024-48218-z (PMC11076638; doi:10.1038/s41467-024-48218-z)
Supplement: Supplementary file 3 — Description of Additional Supplementary Files [file 41467_2024_48218_MOESM3_ESM.pdf]

Description of Additional Supplementary Files

Supplementary Movie 1: Ferroelectric polarization inversion at low magnification of the biasing in-situ TEM system.

Supplementary Movie 2: Ferroelectric polarization inversion at HRTEM mode of the biasing in-situ TEM system.
